# Supplementary material for: Bullous Central Serous Chorioretinopathy: A Rare and Atypical Form of Central Serous Chorioretinopathy. A Systematic Review
Source: Pharmaceuticals (Basel). 2020 Aug 28;13(9):221. doi: 10.3390/ph13090221 (PMC7559580; doi:10.3390/ph13090221)
Supplement: Supplementary file 1 [file pharmaceuticals-13-00221-s001.pdf]

Table 1. Studies included in the systematic review.

Author	Year	Study Design	Sample	Ocular assessments	Outcomes	Level	Strength	Grade
Maggio E	2020	Case report	1	To report a case of bCSCR treated with eplerenone and subsequently with scleral thinning surgery	Two months of oral eplerenone, treatment, were associated with the subretinal fluid Significant increase. Scleral thinning surgery allowed a complete SRF resolution the day after, with no recurrence at six months of follow-up.	5	III	Very low
Aggarwal K	2019	Case report	1	To report a case of bCSCR treated with eplerenone.	Visual acuity improved and SRF resolved after 12 weeks of treatment with 25 mg of oral eplerenone.	5	III	Very low
Ramos-Yau EC	2018	Case report	1	To report a case of bCSCR treated with spironolactone.	Visual acuity improved and SRF resolved after 2 months of treatment with 50 mg of oral spironolactone.	5	III	Very Low
Kowalczuk L	2018	Retrospective case series	3	To investigate the molecular composition of SRF in bCSCR and rhegmatogenous retinal detachment using proteomics and metabolomics.	bCSCR exhibited a distinct molecular profile compared with rhegmatogenous retinal detachment	5	III	Very low
Cebeci Z	2017	Case report	1	To report a case of bCSCR	bCSCR can be misdiagnosed as Harada's disease	5	III	Very low
Venkatesh P	2016	Case report	1	To report a bCSCR case treated with scleral resection and mitomycin C 0.02%	Exudative detachment resolved in 4 months	5	III	Very low
Balaratnasingam C	2016	Retrospective case series	21	To compare qualitative and quantitative imaging characteristics of the choroid, retinal pigment epithelium, and retina in bCSCR versus CSCR	PED are more frequent in bCSCR and usually are hyperreflective. Choriocapillaris and choroid vessels hyperreflectivity, retinal folds and subretinal fibrin are identified more frequently in bCSCR group.	5	III	Low
John VJ	2014	Case report	1	To report a bCSCR case treated internal drainage by vitrectomy along with a scleral buckle placement and silicon oil tamponade	After one year silicon oil was removed and OCT showed no subretinal fluid	5	III	Very low

Lee SB	2013	Case report	1	Bilateral central serous chorioretinopathy with retinal pigment epithelium tears following epidural steroid injection.	Epidural steroids injection could be a risk factor for bCSCR development	5	III	Very low
Kawamura R	2012	Retrospective case series	8	To report treatment of bCSCR with transpupillary thermotherapy	Transpupillary thermotherapy seems to be effective for bCSCR treatment in the short term	5	III	Low
Ng WW	2011	Case report	1	To report half-dose verteporfin photodynamic therapy for treatment of bCSCR.	Three months after photodynamic therapy, there was complete resolution of SRF and bullous retinal detachment.	5	III	Very low
Wykoff CC	2009	Case report	1	To report treatment of bCSCR with reduced fluence verteporfin photodynamic therapy.	Photodynamic therapy can be a therapeutic option.<br>Anti-VEGF Intravitreal injection had no clinical benefit.	5	III	Very low
Barbazetto IA	2009	Case report	1	To report a case of bCSCR complicated by peripheral neovascularization	Focal laser photocoagulation to the active retinal pigment epithelial leaks in the posterior pole resulted in resolution of the bullous detachment and regression of the retinal neovascularization.	5	III	Very low
Kunavisarut P	2009	Retrospective case series	7	To describe clinical features of bCSCR	Early diagnosis of bCSCR prevents the complications from harmful medications as well as unnecessary surgery	5	III	Low
Williams P	2009	Case report	1	A pituitary adenoma was discovered by neuroimaging in a patient with bilateral bCSCR	Bilateral bCSCR can be a rare presentation of pituitary adenoma.	5	III	Very low
Fawzi AA	2006	Retrospective case series	8	To report the rate of bCSCR after solid organ transplantation.	Organ transplantation is a risk factor for bCSCR development	5	III	Low
Kang JE	2006	Case report	1	A patient with bCSCR was treated with vitrectomy and internal drainage of subretinal fluid.	The retina was attached successfully	5	III	Very low
Chen JC	2005	Case report	1	To report a vase of atypical CSCR	Failure to differentiate this condition from inflammatory disease of the retina and choroid may result in inappropriate use of	5	III	Very low

					corticosteroids, leading to exacerbation of the condition and permanent visual loss.			
Chan WM	2004	Retrospective case series	2	To report two cases of bCSCR presenting with peripheral retinal neovascularization	The final visual prognosis is, in general, related to the duration of the retinal detachment, healthiness of the RPE, and any formation of submacular fibrosis.	5	III	Low
Chen HC	2003	Case report	1	To report treatment of bCSCR with pars plana vitrectomy associated with trans-scleral drainage	Immediate retinal reattachment without the need of retinotomy	5	III	Very low
Otsuka S	2002	Consecutive observational case series	25	Understanding of the long-term course and visual outcome of a severe variant of central serous chorioretinopathy.	Exudative chorioretinal lesions are self-limited or respond to photocoagulation. Recurrence is common.	4	III	Low
Adan A	2001	Case report	1	To report a case bCSCR treated with pars plana vitrectomy combined with trans-scleral drainage	Immediate retinal reattachment without the need of retinotomy	5	III	Very low
Sahu DK	2000	Retrospective case series	13	To compare argon laser photocoagulation versus no observation	Laser treatment did not offer additional benefit over the natural course of the disease	5	III	Low
Uyama M	1999	Retrospective case series	45	To clarify the pathophysiology of multifocal posterior pigment epitheliopathy, or bullous retinal detachment	Involvement of the RPE may be secondary, and then the disease becomes manifest with exudative retinal detachment.	5	III	Low
Sharma T	1998	Retrospective case series	29	To report treatment of bCSCR with argon photocoagulation	Retinal reattachment was obtained in all cases and visual acuity improved >2 lines in 68.9% of patients.	5	III	Low
Yang CM	1998	Case report	1	Toi report a misdiagnosis of bCSCR	Suspect bCSCR in patients with exudative retinal detachment, as the use of steroids is usually not only unnecessary but can also be harmful.	5	III	Very low
Hooymans JM	1998	Case report	1	To report an atypical form of CSCR in one patient assuming systemic corticosteroids	Systemic steroids could be a risk factor for bCSCR development	5	III	Very low
Gass JD	1995	Case report	3	To report an atypical form of CSCR in three patients assuming systemic corticosteroids	Systemic steroids could be a risk factor for bCSCR development	5	III	Low

Gass JD	1991	Case series	6	To report an atypical form of CSCR in six pregnant women	Pregnancy could be a risk factor for bCSCR development	5	III	Low
Schreiber JB	1989	Case report	1	Crohn's disease complicated by bCSCR	Another ophthalmologic complication of inflammatory bowel disease.	3	III	Very low
Akiyama K	1987	Retrospective case series	3	To report three cases of bCSCR examined during a follow-up ranging from 2 to ten years.	Large areas of RPE degeneration were observed in all patients, Besides, one showed a retinal neovascularization.	3	III	Low
Mazzuca DE	1986	Case report	1	To characterize bCSCR	bCSCR is frequently misdiagnosed and subsequently treated improperly	3	III	Very low
Kaneko E	1985	Case report	1	Ulcerative colitis complicated by idiopathic central serous chorioretinopathy with bullous retinal detachment	Steroid therapy was not effective on the ocular disorder.	5	III	Very low
Gass JD	1973	Case report	1	To describe the first case of bCSCR in literature	An atypical form of CSCR in which a subretinal exudation at the posterior pole is associated with an inferior exudative retinal detachment	5	III	Very low

bCSCR: bullous central serous chorioretinopathy, SRF: subretinal fluid, VEGF: vascular endothelial growth factor.
